# Chromosomal level genome assembly of medicinal plant *Sophora flavescens*

**DOI:** 10.1038/s41597-023-02490-8

**Published:** 2023-08-29

**Authors:** Zhipeng Qu, Wei Wang, David L. Adelson

**Affiliations:** 1 Zhendong Center, Department of Molecular and Biomedical Sciences, The University of Adelaide, Adelaide, 5005 Australia; 2 Beijing Zhendong Research Institute, Shanxi Zhendong Pharmaceutical Co Ltd, Beijing, 10587 China; 3 Shanxi Provincial Key Laboratory of Functional Food with Homology of Medicine and Food, Department of Pharmacy, Changzhi Medical College, Changzhi, 046012 China; 4 South Australian Museum, Adelaide, 5000 Australia

**Keywords:** Plant sciences, Computational biology and bioinformatics

## Abstract

*Sophora flavescens* is a medicinal plant in the genus *Sophora* of the Fabaceae family. The root of *S. flavescens* is known in China as Kushen and has a long history of wide use in multiple formulations of Traditional Chinese Medicine (TCM). In this study, we used third-generation Nanopore long-read sequencing technology combined with Hi-C scaffolding technology to *de novo* assemble the *S. flavescens* genome. We obtained a chromosomal level high-quality *S. flavescens* draft genome. The draft genome size is approximately 2.08 Gb, with more than 80% annotated as Transposable Elements (TEs), which have recently and rapidly proliferated. This genome size is ~5x larger than its closest sequenced relative *Lupinus albus L*. . We annotated 60,485 genes and examined their expression profiles in leaf, stem and root tissues, and also characterised the genes and pathways involved in the biosynthesis of major bioactive compounds, including alkaloids, flavonoids and isoflavonoids. The assembled genome highlights the very different evolutionary trajectories that have occurred in recently diverged Fabaceae, leading to smaller duplicated genomes.

## Background & Summary

The *Sophora* genus is a member of the Fabaceae family, that includes more than 52 species, 19 varieties and 7 forms distributed mainly in Asia, Oceania and the pacific islands^1^. More than fifteen species in the genus *Sophora* have been used in Traditional Chinese Medicines (TCMs) for hundreds of years^2^. The root of *Sophora flavescens*, which is known as “Kushen” in China, has been widely used for the treatment of symptoms such as fevers, dysentery, jaundice, vaginal itching with leukorrhagia, abscesses, carbuncles, enteritis, leukorrhea, pyogenic infections of the skin, scabies, swelling, and pain in different TCM formulations^3^. The extracts of *S. flavescens* are mainly used in compounds or as decoctions with other herbal products and are taken orally. However, the characterisation of chemical profiles of *S. flavescens* extracts and improved manufacturing techniques, such as Good Manufacturing Practices (GMP) that comply with guidelines from State Food and Drug Administration (SFDA) in China, have led to the approval of injectable formulations for clinical treatment of cancer and infectious diseases^3^.

One *S. flavescens* based injection is Compound Kushen injection (CKI, also known as Yanshu injection). CKI is extracted from *S. flavescens* and another medicinal plant Baituling (*Heterosmilax yunnanensis*) using modern, standardised GMP. It is a State Administration of Chinese Medicine-approved TCM formula used for the clinical treatment of various types of cancers in China. Multiple evidence-based bioactive compounds, most of which are from *S. flavescens*, have been characterised from CKI^4^. Studies from *in vitro* or *in vivo* experiments have shown that CKI can inhibit cancer cell proliferation, induce apoptosis and reduce cancer-associated pain^5,6^. It is one of the approved drugs in the National Basic Medical Care Insurance Medicine Catalogue for cancer treatment in many provinces of China. The pharmaceutical market of CKI has transformed the production of *S. flavescens* from traditional wild collection to commercial-scale field cultivation. However, there is little genomic information available for *S. flavescens*, which has greatly hindered the breeding of *S. flavescens* and characterisation of its bioactive compounds.

Fabaceae (or Leguminosae) is a large and diverse flowering plant family including 6 subfamilies^7^. Of the 6 subfamilies, Papilionoideae is the largest one and includes most agriculturally important legumes, such as soybean (*Glycine max*) and pea (*Pisum sativum*). These grain legumes are important sources of plant-derived proteins, and are important alternatives to animal-derived proteins in food^8^. Therefore, the genome sequencing and assembly of Fabaceae family species has focused on cultivated Papilionoideae legumes^9^. *S. flavescens* is a wild Papilionoideae legume from the early-diverged Genistoid clade. The key synapomorphy of the Genistoid clade is the production and accumulation of quinolizidine alkaloids (QAs), which play essential defence roles in the adaption to wild environments^10^. The chemosystematic analysis of taxonomic patterns of secondary metabolites in Genistoid tribes has provided phylogenetic clues for the characterisation of their relative position in the evolution of papilionoid legumes^11^. Recently, the reference genome of one of the important Genistoids, lupin species, has been sequenced and this has provided genetic resources for understanding the biosynthesis of secondary metabolites in the Genistoid clade^12^. The characterisation of genes and pathways involved in the biosynthesis of QAs in lupin is important for the domestication of lupin as the QA content in lupin seeds must be under the industry safety threshold (0.02%) for food purposes^13^. In contrast to lupin species, secondary metabolites, particulary QAs in *S. flavescens*, are important for its medicinal use in the pharmaceutical industry. *S. flavescens* and lupin species share similar QA biosynthetic pathways, while producing different end compounds, matrine and oximatrine for *S. flavescens* and lupanine for lupin species. Therefore, the *S. flavescens* reference genome is important for further understanding of the regulatory and biosynthetic pathways of QAs in Genistoids. In addition, the comparative genomics analysis between *S. flavescens* and lupin species will also provide insights to the molecular evolution of leguminosae species.

**Fig. 1:**
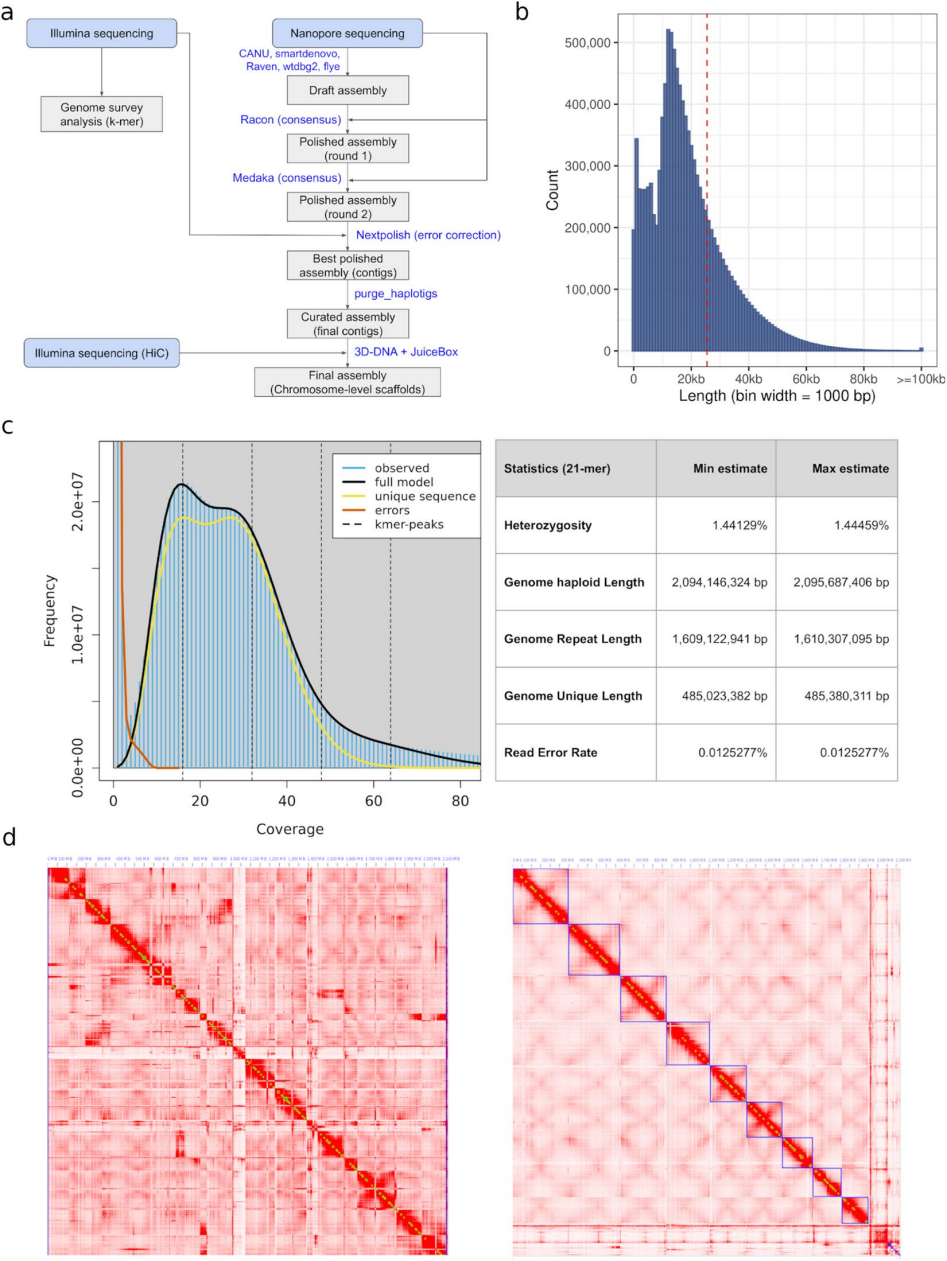
Chromosomal level assembly of *S. flavescens* genome. (**a**) Genome assembly flowchart. (**b**) Length distribution of Nanopore ONT reads. (**c**) Genome survey analysis. (**d**) Contact map of Hi-C interaction for assemblies before scaffolding (left) and after scaffolding (right).

In this study, we completed a chromosomal level draft genome assembly of *S. flavescens* by implementing and comparing multiple assembly strategies using sequencing data from multiple platforms (Fig. 1a). From the best assembly we predicted *ab initio* 60,485 genes and annotated ~83% of assembled genome regions as transposable elements (TEs). Comparative phylogenomic analyses of 16 legumes and 9 outgroup species indicated that *S. flavescens* has the highest rate of gene expansion of the analysed legumes and has followed a strikingly different genome evolution trajectory compared to other legumes, including its closest relative *Lupinus albus L*. . We also characterised the genes/proteins involved in the biosynthesis of two major categories of bioactive compounds, alkaloids and flavonoids/isoflavonoids, confirming the high quality of this *S. flavescens* draft genome assembly. This genome assembly will be a valuable genomic resource for understanding the biosynthesis of bioactive compounds in *S. flavescens*, for plant breeding and for the molecular characterisation of geographically different subspecies of *S. flavescens*.

## Methods

### Sample collection and sequencing

#### Plant materials

One individual *S. flavescens* plant grown in the plantation of Pingshun County, Shanxi, China (36.2001° N, 113.4361° E) was collected as the source of genomic DNAs or total RNAs. All libraries and sequencing were carried out by Benagen (Wuhan, China). The detailed protocols are as follows.

#### Nanopore sequencing

Young fresh leaves were collected and immediately used for high-quality genomic DNA isolation with the CTAB (cetyltrimethylammonium bromide) method. The quality of isolated genomic DNAs was examined using agarose gel electrophoresis, and then high-quality genomic DNA was randomly fragmented using a Megaruptor (Diagenode, NJ, USA). High molecular weight (HMW) DNA fragments were selected using the BluePippin system (Sage Science, USA), and then prepared and ligated with adapters using Nanopore SQK-LSK109 (Oxford Nanopore technologies, USA). Ligated DNA libraries were examined again using a Qubit and loaded on to Nanopore Flow cells R9.4, and sequenced on the PromethION platform (Oxford Nanopore technologies, USA).

In total, ~11 million Nanopore ONT long reads (approximately 222 Gb) were generated for the *de novo* whole genome assembly. The N50 for the nanopore reads was ~25 Kb, and the longest raw read had a length of 219 Kb (Fig. 1b).

#### Illumina sequencing

Genomic DNA from the young fresh leaves of the same plant was isolated using the same methods as for the Nanopore sequencing. To generate small fragments for sequencing, high-quality genomic DNA was randomly fragmented using a Covaris ultrasonicator (Covaris, USA). Illumina sequencing libraries were constructed using the Truseq nano DNA HT library preparation kit (Illumina, USA) with targeted insertion size of 350 bp. Purified libraries were loaded and sequenced on the Illumina NovaSeq. 6000 platform (Illumina, USA).

In total, we obtained ~1,500 million Illumina short reads (approximately 226 Gb), which were used for genome survey analysis of *S. flavescens* and error correction in genome assembly.

#### Hi-C library preparation and sequencing

The Hi-C library was prepared using a modified method according to the protocol from Ramani *et al*.^14^. In summary, young fresh leaves from the same plant were collected, and fixed using formaldehyde. Then fixed tissues were homogenised and centrifuged to isolate nuclei. Cross-linked chromatin was digested with DpnII and labelled with Biotin, and then was ligated using T4 DNA ligase. DNA was purified and examined using agarose gel electrophoresis. Finally, the library was prepared and sequencing was carried out according to the above-mentioned Illumina sequencing protocol.

About 1,600 million Hi-C reads (approximately 242 Gb) were obtained for scaffolding in genome assembly.

#### Transcriptome sequencing

Total RNA from leaves, stems and roots of the same plant was isolated using the TRIZOL method, and libraries were prepared using the Illumina TruSeq RNA library Prep kit. Sequencing was carried out on the Illumina NovaSeq. 6000 platform (Illumina, USA).

### Genome survey analysis

Adaptor and low quality sequences in Illumina raw reads were trimmed using Trimmomatic (v0.39)^15^ with the following parameters: LEADING:3 TRAILING:3 MINLEN:36. The frequencies of 21-mers in clean reads were calculated using jellyfish (v2.3.0)^16^ with the following parameters: -C -m 21–min-qual-char = ?. Genome survey analysis was carried out using GenomeScope (v1.0)^17^ with the following settings: k-mer_length = 21 read_length = 300.

The genome survey analysis from the 21-mer frequency distribution of Illumina reads indicated that the *S. flavescens* genome is diploid, and gave a haploid genome size of approximately 2.09 Gb. It has a relatively high level of heterozygosity (~1.4%) and very high abundance of repetitive elements (~80%) (Fig. 1c).

### Error correction of Nanopore raw reads

Two different methods were used to error-correct Nanopore raw reads. The error correction module in CANU (v2.0) was used to self-correct the Nanopore raw reads by building consensus sequences based on long reads alone with the following parameters: genomeSize = 2.1 g corMinCoverage = 2 corOutCoverage = 200 “batOptions = -dg 3 -db 3 -dr 1 -ca 500 -cp 50” correctedErrorRate = 0.12 corMhapSensitivity = normal ovlMerThreshold = 500 -nanopore^18^. FMLRC was used to error-correct the Nanopore raw reads using Illumina sequencing reads with default settings^19^.

### *S. flavescens* draft genome assembly

To obtain a high-quality reference genome, we used 17 different assembly strategies (Supplementary Table 1). The initial strategy was to use the CANU-only (v2.0) pipeline. After the error-correction of Nanopore reads using the CANU “correct” module, we used the CANU “trim” module to remove low quality regions in error-corrected reads. The genome was then assembled using the CANU “assemble” module with the following parameters: genomeSize = 2.1 g corMinCoverage = 2 corOutCoverage = 200 “batOptions = -dg 3 -db 3 -dr 1 -ca 500 -cp 50” correctedErrorRate = 0.12 corMhapSensitivity = normal ovlMerThreshold = 500 -nanopore^18^. In addition, we also tried four other assemblers, including Raven (v1.1.10)^20^, SMARTdenovo (v1.0)^21^, wtdbg2 (v1.1)^22^ and Flye (v2.7.1)^23^ on four different input datasets respectively. The first input dataset includes all nanopore raw reads (named as “raw_all”). The second input dataset is a subset of the first dataset, including only raw reads longer than the N50 of all raw reads (named as “raw_N50”). The third input dataset includes error-corrected Nanopore reads using CANU (named as “canu_ec”). And the fourth input dataset includes error-corrected reads longer than the N50 of all error-corrected reads using FMLRC (v1.0.0) (“fmlrc_N50”)^19^. Three polishing steps were carried out for draft genomes, including: a, four rounds of polishing using racon (v1.4.16)^24^ based on nanopore reads with the following parameters: -m 8 -x -6 -g -8 -w 500; b, one round of polishing using medaka (v1.0.3) (Nanopore technologies) based on nanopore reads with the following parameters: -m r941_prom_high_g360 -b 1000; c, two rounds of polishing using nextpolish (v1.2.4)^25^ based on Illumina reads with default settings. Haplotigs in the polished draft genomes were purged using purge_haplotigs (v1.1.1)^26^ following instructions in the documentation.

Comparison of draft genome assemblies from these 17 different assembly strategies indicated that the draft assembly achieved by using CANU for both error-correction and assembling steps had the longest contig, more than 15 Mb long (Supplementary Table 1). The assembly from CANU error-corrected reads along with those from two other assemblers (“Flye + Canu_ec” and “SMARTdenovo + Canu_ec”) had much longer N50s (longer than 600 Kb) compared to other strategies that gave relatively low numbers of contigs (Supplementary Table 1). With respect to genome size, the CANU-only strategy generated a much larger genome than the other two high contiguity strategies, however, the assessment using BUSCO (v4.1.4)^27^ with lineage “fabales_odb10” yielded many more duplicated otholologs from the CANU-only strategy compared to the other two large contig strategies, indicating the presence of many haplotigs in the CANU assembly (Supplementary Table 1). After haplotig removal, the CANU-only assembly had a genome size of ~2.08 Gb with an N50 longer than 2 Mb, which was much longer than the N50s from other strategies (Table 1). After considering all the assembly statistics, we selected the CANU assembly as the optimal draft genome for subsequent scaffolding, annotation and analysis.

**Table 1:** Statistics of contigs and scaffolds for genome assembly.

Statistics	Contigs	Scaffolds
Total size (bp)	2,073,438,938	2,075,133,938
Number of sequences	3,865	4,353
Mean length (bp)	536,465	476,714
Longest sequence (bp)	16,871,262	299,095,550
shortest sequence (bp)	435	435
Number of Ns (bp)	803	1,695,803
Number of gaps	0	4,144
N50 (bp)	2,601,763	233,466,755
BUSCO (C)	91.4%	91.3%
BUSCO (S)	84.0%	86.7%
BUSCO (D)	7.4%	4.6%
BUSCO (F)	2.0%	2.0%
BUSCO (M)	6.6%	6.7%

Then, contigs in the draft genome were scaffolded using 3D-DNA (v4.1.4)^28^ with the Hi-C sequencing reads. Scaffolds were then manually curated using Juicebox (v1.13.01)^29^ following the guidelines in the documentation. We obtained nine chromosomal level scaffolds (Fig. 1d) along with 4,344 un-anchored scaffolds (Table 1). These nine scaffolds most likely correspond to the nine chromosomes of *S. flavescens* (Fig. 1d)^30^.

### *De novo* annotation of genes and TEs in the *S. flavescens* draft genome

#### Transcriptome

Illumina RNA-Seq raw reads from leaf, stem and root tissues were trimmed using Trimmomatic (v0.39) with the following parameters: LEADING:3 TRAILING:3 MINLEN:36. For *de novo* transcriptome assembly, clean reads from three tissues were merged and assembled into transcripts using StringTie (v2.1.4) with default settings^31^. After the genome was assembled, the genome alignment of RNA-Seq data were carried out using STAR (v2.7.8a)^32^ with the following parameters:–outSAMstrandField intronMotif–outSAMattributes All–outFilterMismatchNmax 10–outFilterMismatchNoverLmax 0.03–outFilterMultimapNmax 5–alignIntronMax 10000.

#### *Ab initio* gene annotation

**Fig. 2:**
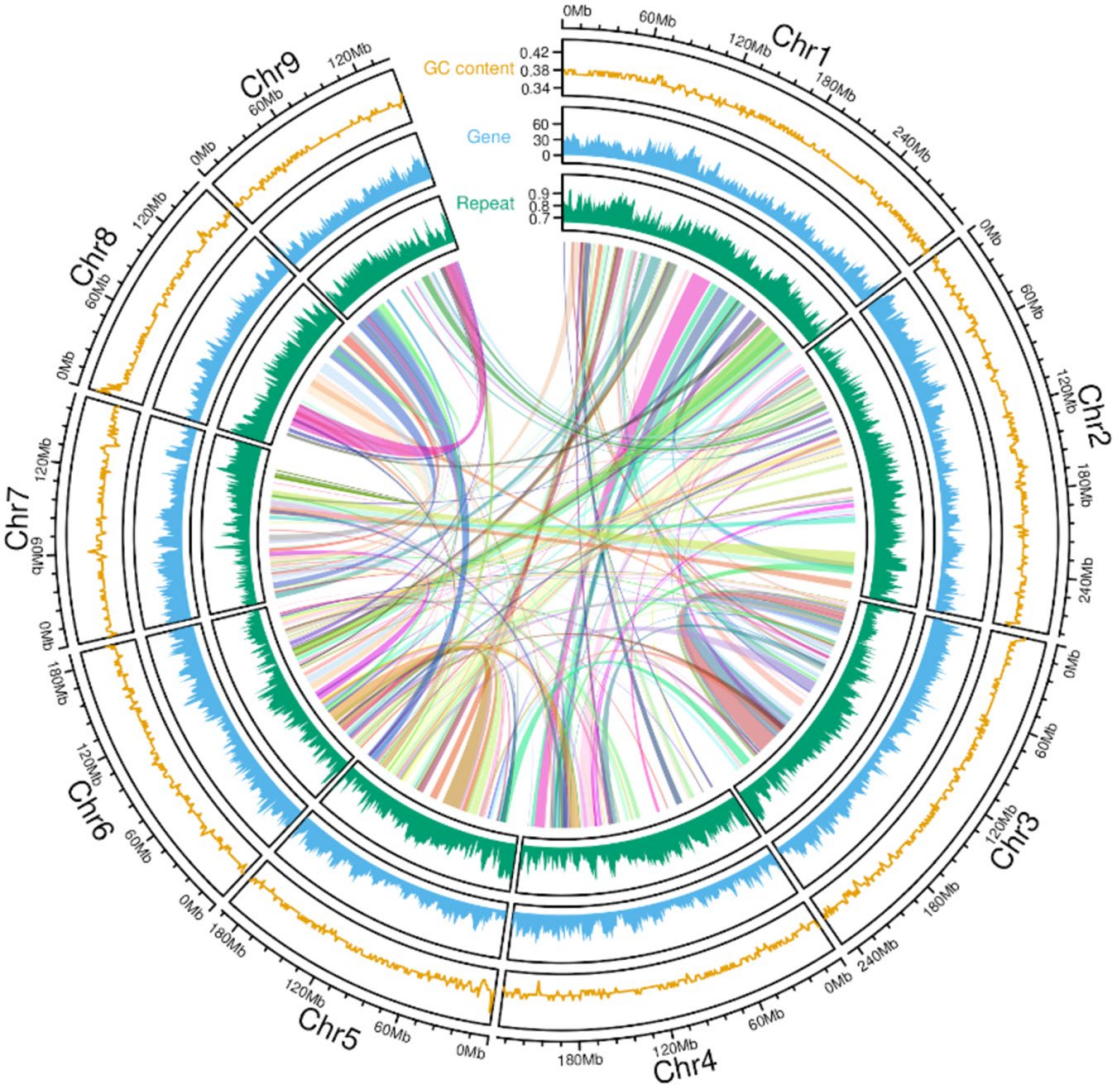
Circos plot showing the genomic distribution of genes and TEs. The Y axis for the track of GC content represents the coverage of GC bases in 100Kb bins. The Y axis for the gene track represents the number of genes in 100Kb bins. The Y axis for the repeat track represents the ratio of bases covered by TEs in 100Kb bins.

The *ab initio* gene annotation of *S. flavescens* genome was carried out using Maker (v3.01.03)^33^. Gene models trained with Augustus (v3.2.3)^34^, as well as *de novo* assembled transcripts from three tissues, were used as transcription evidence to support gene prediction by Maker. Three rounds of Maker annotation were carried out, and only gene models with AED score < 0.5 and protein length > 10 were used in each round of annotation. In total, 60,485 genes were identified from the assembled *S. flavescens* reference genome (Fig. 2).

**Fig. 3:**
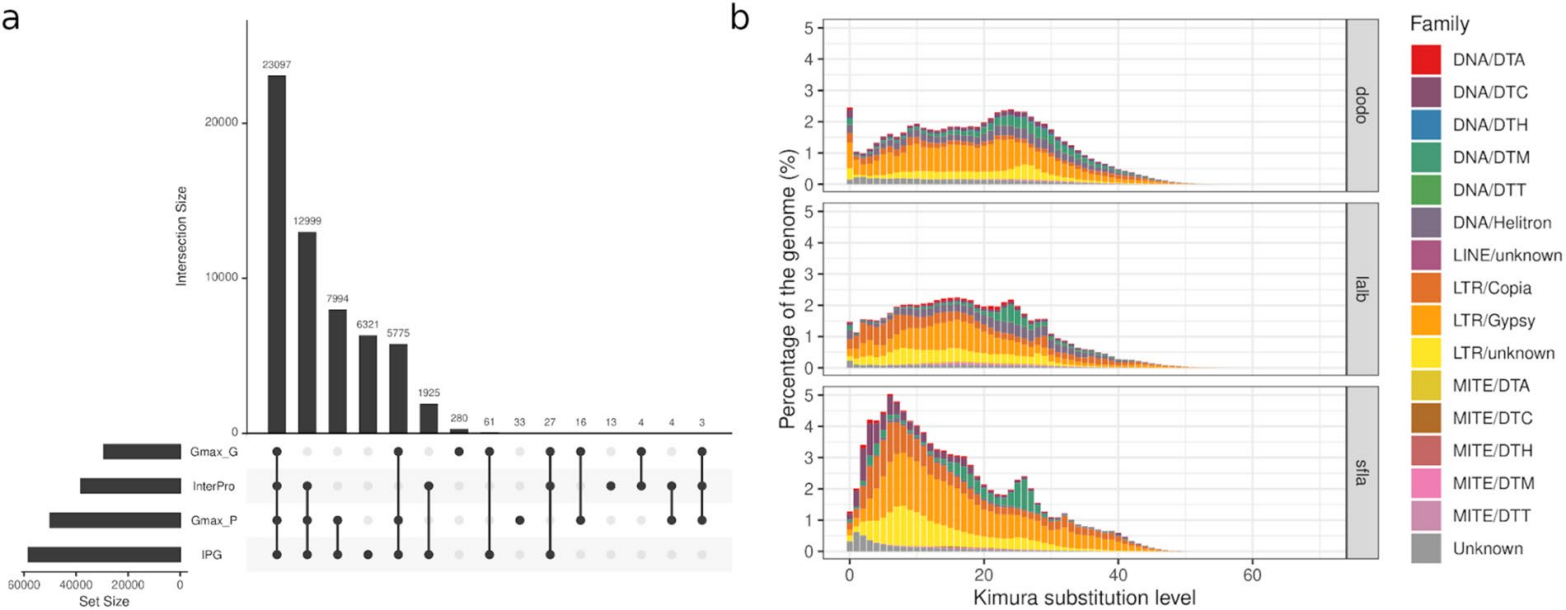
*Ab initio* gene and TE annotation for *S. flavescens* draft genome. (**a**) Number of genes annotated by different databases. (**b**) Divergence (Kimura substitution) plot of different TE families in *S. flavescens* (sfla), *L. albus L*. (lalb), and *D. odorifera* (dodo).

We then did functional annotation for predicted genes/proteins using BLAST with a threshold e-value < 1e-3 against four well-curated databases, including all Fabales proteins from NCBI IPG (Identical Protein Groups), InterPro protein families, Ensemble *Glycine max* reference genes and proteins^35–38^. This resulted in 58,552 *S. flavescens* genes (96.8%) annotated on the basis of at least one database (Fig. 3a).

TEs in the *S. flavescens* genome were predicted and annotated using the pipeline of Extensive *de novo* TE Annotator (EDTA, v1.9.7)^39^. Our *ab initio* prediction of repeats in the *S. flavescens* genome revealed a total of 2,401,867 TEs, accounting for 83.06% of the assembled genome (Supplementary Table 2, Fig. 2). The majority of predicted TEs are long terminal repeat retrotransposons (LTRs), with *Gypsy*, *Copia* and unknown LTRs comprising 30.51%, 15.12% and 17.08% of the genome respectively, for a total of more than 60% of the assembled genome (Supplementary Table 2).

To compare the distribution of TE families with respect to divergence, we first re-annotated TEs in *L. albus L*. and *Dalbergia odorifera* using EDTA, and then calculated the Kimura substitution levels of annotated repeats using the script “createRepeatLandscape.pl” from RepeatMasker. The Kimura substitution level of different TE families in *S. flavescens* revealed that the majority of *Mutator* DNA transposons (DNA/DTM) showed high Kimura substitution rate (20–30%), indicating that these are ancient repeats contributing to ancestral legume genome evolution. This is also supported by the similarly high Kimura substitution level (20–30%) of DNA/DTM in two close relatives, *L. albus L*. and *D. odorifera* (Fig. 3b). However, compared to *L. albus L*. and *D. odorifera*, the *S. flavescens* genome contains many more “younger” LTRs (Kimura substitution level less than 10%), including *Copia*, *Gypsy* and unknown LTRs as well as CACTA DNA transposons (DNA/DTC), indicating that there was a relatively recent TE expansion in *S. flavescens* mainly driven by LTRs. We believe this accounts for the huge genome size difference between these two closely related species, *S. flavescens* and *L. albus L*. (~450 Mb)^12^.

### Phylogenomics

Orthologs between *S. flavescens* and 25 other plant species, including 16 legumes and 9 outgroups (Supplementary Table 3), were identified using OrthoFinder (v2.5.4)^40^ with all primary proteins in each species. The phylogenetic tree was constructed with IQ-TREE (v1.6.12)^41^ using “JTT + F + R5” as the best-fit model based on alignment blocks of single-copy orthologs obtained from OrthoFinder. Divergence times in the phylogeny were estimated using r8s (v1.81)^42^ with time constraints for the most recent common ancestors (MRCA) of nodes between *Nelumbo nucifera* (Nnuc) and *Vitis vinifera* (Vvin) of (122.59–126.00 MYA), between *Lupinus angustifolius* (Lang) and *Glycine max* (Gmax) of (45.42–62.84 MYA), between *Lotus Japonicus* (Ljap) and *Medicago truncatula* (Mtru) of (36.46–53.58 MYA)^43^.

**Fig. 4:**
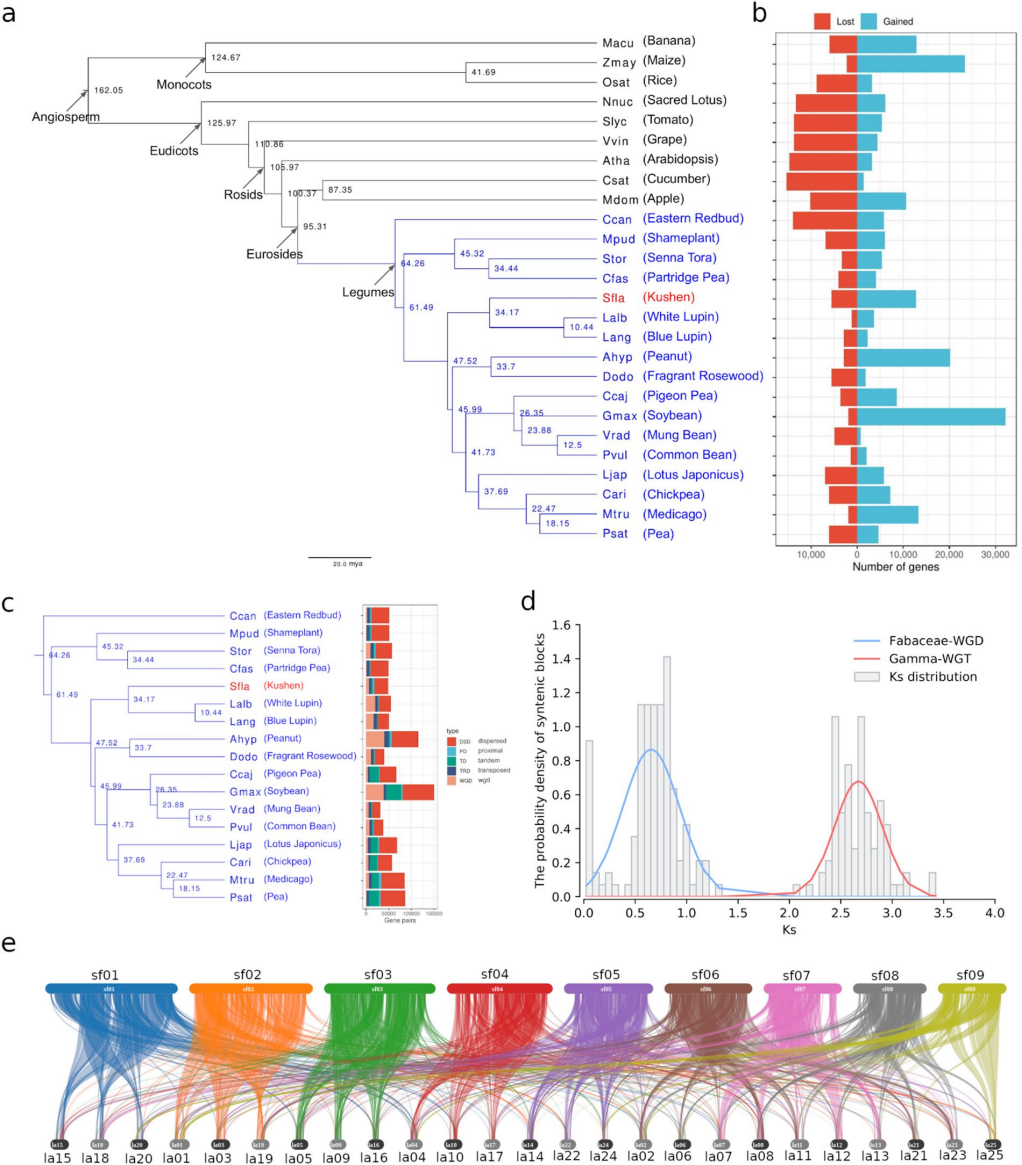
Phylogenomics analysis of *S. flavescens*. (**a**) Phylogeny of *S. flavescens* with other 16 legumes and 9 outgroups. (**b**) number of expanded/contracted genes. (**c**) Numbers of different types of duplicated gene pairs in different legumes. (**d**) Ks distribution of syntenic blocks characterised based on WGD genes in *S. flavescens* genome. (**e**) Gene syntenic blocks between *S. flavescens* chromosomes (top) and *Lupinus albus L*. chromosomes (bottom). Colour coding represents different chromosomes in *S. flavescens*.

In summary, we identified 46,397 orthogroups from these 26 species, and 15,576 of them have at least one *S. flavescens* gene. We then constructed a phylogeny from these orthologs that showed that the core genistoides, *S. flavescens* and *Lupinus* diverged from other cultivated grain legumes, mostly Phaseoloides (e.g. soybean), Galegoids (e.g. pea, Medicago, chickpea) and Dalbergoids (e.g. peanut) ~47 million years ago (MYA), followed by the divergence of *S. flavescens* and *Lupinus* ~34 MYA (Fig. 4a)^44^.

Based on the identified orthologs and the phylogenetic tree, gene expansion and contraction analysis was carried out using CAFE (v4.2.1) following the CAFE manual^45^. This analysis showed that *S. flavescens* also has undergone more gene family expansion than contraction (Fig. 4b). Furthermore, in legumes it has the highest average gain of 4.29 genes/family in 2,965 expanded families (Supplementary Table 4).

Whole genome duplication (WGD) analysis was carried out using DupGen_finder with *N. nucifera* as the outgroup^46^. We found 27,809 duplicated genes that are part of 36,406 duplicated gene pairs (Fig. 4c, Supplementary Table 5). Synonymous nucleotide substitution rates (Ks) for identified WGD pairs in *S. flavescens* and other 16 legumes were calculated using ParaAT (Version: 2.0, release Oct. 4, 2014)^47^. Ks peaks were inferred by fitting a Gaussian Mixture Model (GMM) to Ks distributions according to Qiao *et al*.s’ method^46^. The Ks distribution of *S. flavescens* revealed two peaks (Fig. 4d), indicating two potential WGD/WGT events during *S. flavescens* evolution. It had previously been shown that there was one Fabaceae WGD (peak at ~0.8) after the ancestral *γ* WGT event (peak at ~2.7)^46^. Our results are consistent with these two previously reported WGD/WGT events which we detected in *S. flavescens*.

Gene synteny blocks between *S. flavescens* and *L. albus L*. were identified using MCScanX (v1)^48^ and visualised using the online tool SynVisio^49^. The gene synteny map indicated that almost every *S. flavescens* pseudo-chromosome has three repeated, independent chromosomal level counterparts in the *L. albus L*. genome (Fig. 4e).

### Identification and characterisation of genes involved in the biosynthesis of bioactive compounds in *S. flavescens*

#### Alkaloids

**Fig. 5:**
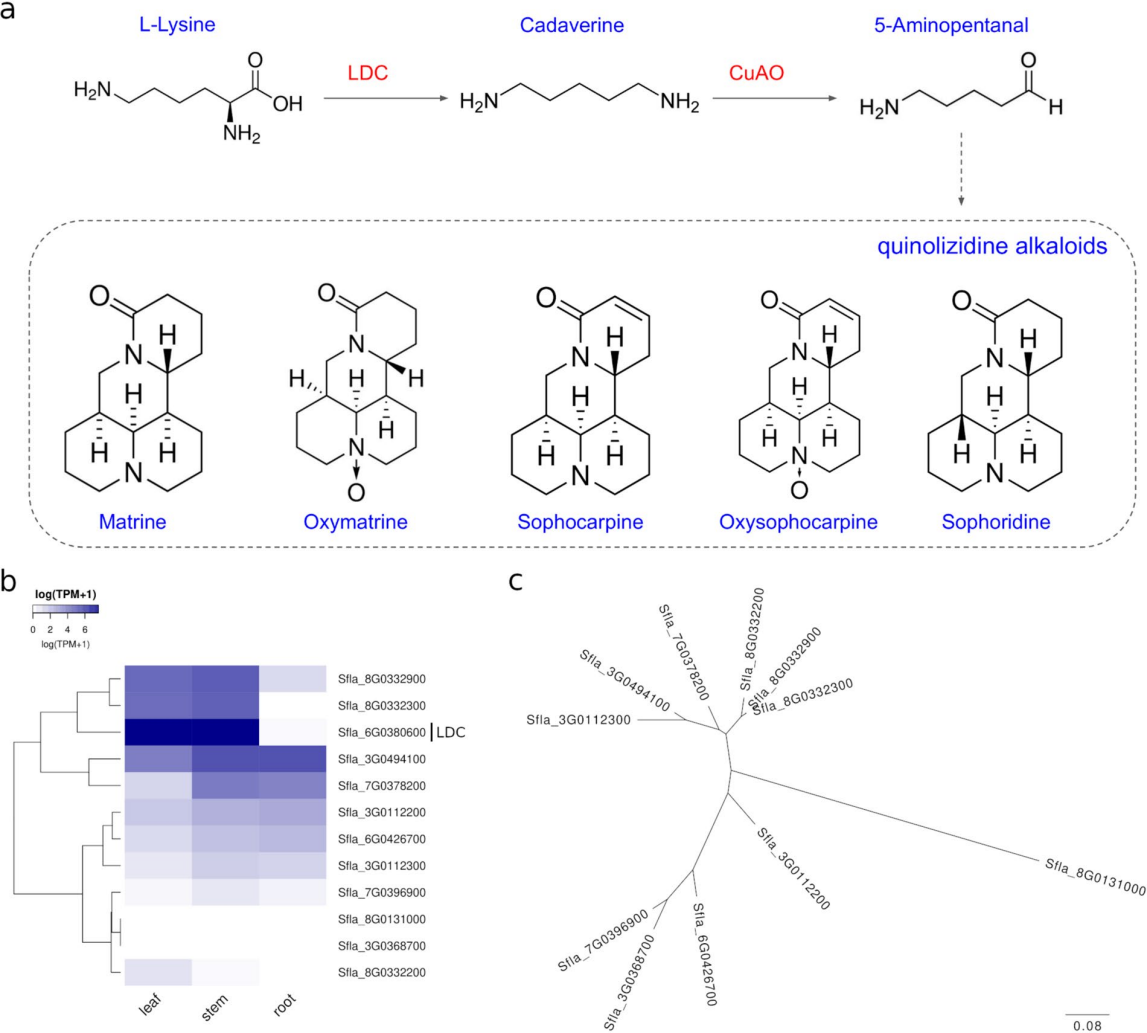
Characterisation of genes involved in the biosynthesis of alkaloids in *S. flavescens*. (**a**) Biosynthesis process of major alkaloids in *S. flavescens*. (**b**) Expression profile of genes involved in the biosynthesis of alkaloids in *S. flavescens*. (**c**) Phylogenetic tree of *S. flavescens* candidate genes encoding CuAO.

Most bioactive alkaloids from *S. flavescens*, such as oxymatrine and matrine, are quinolizidine alkaloids (QAs). QAs are defensive secondary metabolites produced by plants from the genistoid clade of legumes to protect against insect pests^50^. The core protein in the biosynthesis process of QAs is Lysine/ornithine decarboxylase (LDC), which converts L-lysine to Cadaverine through decarboxylation (Fig. 5a)^50^. To characterise LDC gene, the protein sequence of LDC from *S. flavescens* was retrieved from GenBank (accession number: AB561138.1). Gene/Protein of LDC in our assembled *S. flavescens* draft genome was identified by sequence similarity search using the retrieved LDC protein against all our predicted proteins using BLASTP with a cutoff value of e-value < 1e-3. The top hit of the similarity search in our predicted proteins was annotated as LDC. One copy of the LDC gene (Sfla_6G0380600) was characterised from our assembled *S. flavescens* genome. We also examined the expression levels of the LDC gene in three different tissues using RSEM (v1.2.30) to calculate the normalised expression values (TPM, Transcripts Per Million) based on the transcriptome data^51^, and abundant expression was observed in leaves and stems but not in roots (Fig. 5b). This is consistent with a previous report showing that QAs are mainly synthesised in the green parts of plants^52^. The QAs in roots are more likely accumulated by translocation from leaves and stems through phloem^53^.

Another key protein in this biosynthesis process is Copper amine oxidase (CuAO), which oxidises Cadaverine to 5-Aminopentanal^50^. In Arabidopsis, ten genes from the CuAO gene family have been characterised^54^. In our assembled *S. flavescens* genome, eleven genes that potentially encode CuAOs were identified, indicating that they might be from the same CuAO gene family. Five CuAO candidate genes were more highly expressed in stems and roots and two CuAO candidate genes (Sfla_8G0332300 and Sfla_8G0332900) were more highly expressed in leaves and stems (Fig. 5b). We then performed phylogenetic analysis for CuAO genes using web-based ClustalW2 and Simple Phylogeny tools in EMBL_EBI^55^. We found that the two genes that are highly expressed in leaves and stems (Sfla_8G0332300 and Sfla_8G0332900), together with another leaf-only expressed gene Sfla_8G0332200, are more closely related than other CuAO candidates, indicating that they might be CuAOs involved in *S. flavescens* alkaloid biosynthesis (Fig. 5c).

#### Flavonoids

**Fig. 6:**
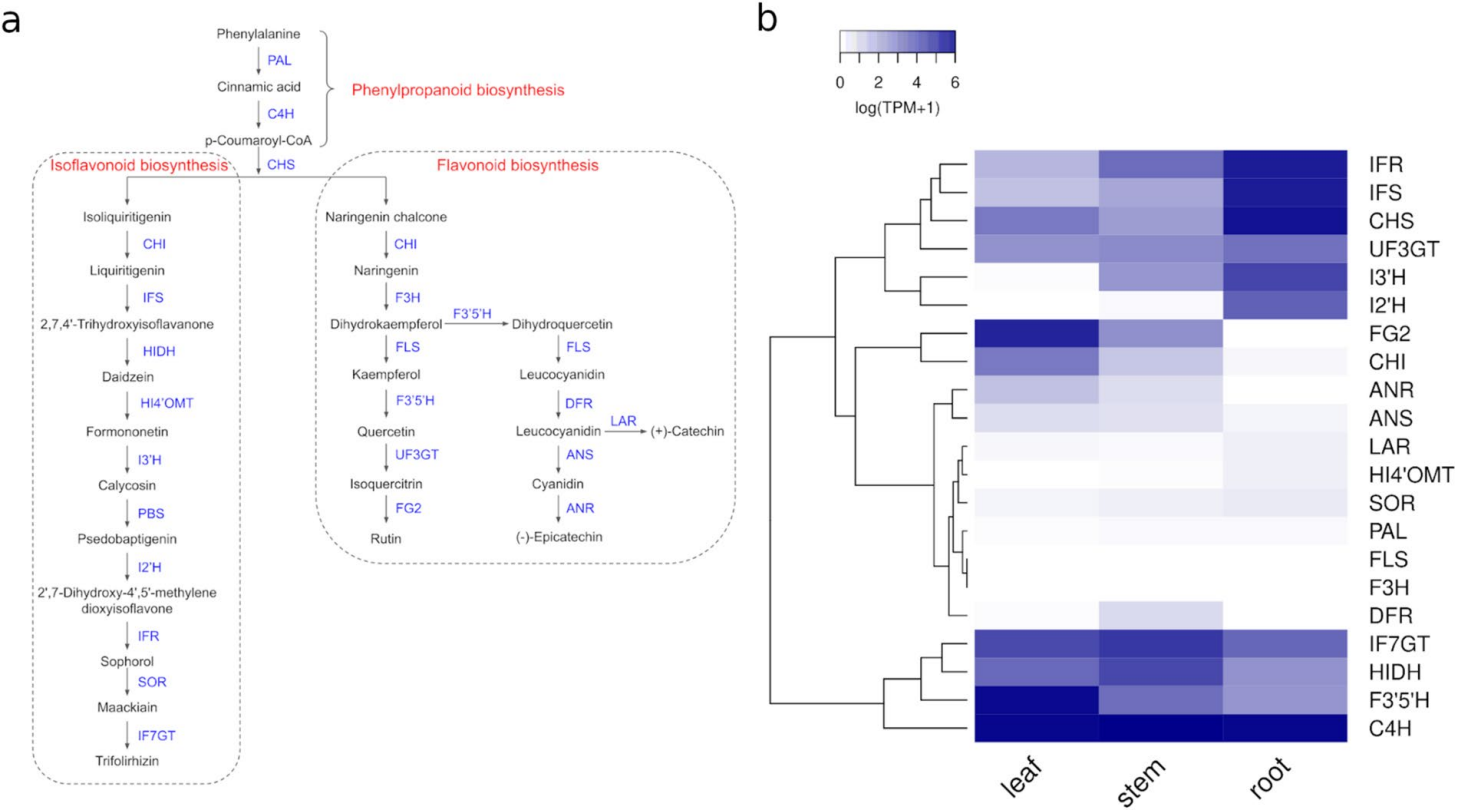
Characterisation of genes involved in the biosynthesis of flavonoids/isoflavonoids in *S. flavescens*. (**a**) Biosynthetic pathways for flavonoids/isoflavonoids. (**b**) Expression profile of genes involved in the biosynthesis of flavonoids/isoflavonoids in *S. flavescens*.

There are 25 key genes involved in the biosynthesis of isoflavonoids and flavonoids in *S. flavescens* based on KEGG pathways and literature (Fig. 6a). Protein sequences of 21 of these 25 genes from either lupin or soybean could be retrieved from GenBank (Supplementary Table 6). Isoflavonoids and flavonoids biosynthesis related genes in *S. flavescens* were annotated with a similarity search of retrieved proteins against our predicted proteins using BLASTP with a cutoff value of e-value < 1e-3. From our assembled draft genome, we were able to successfully characterise all these 21 key genes involved in the biosynthesis of different flavonoids or isoflavonoids (Supplementary Table 6). The expression profile of these 21 genes in three *S. flavescens* tissues showed that several genes were highly expressed in roots, and fewer genes were highly expressed in stems. However, some genes were only expressed in leaves and stems (Fig. 6b).

## Data Records

All raw sequencing data used for genome assembly and analyses have been deposited into Sequence Read Archive database of NCBI and can be accessed according to Bioproject: PRJNA973122 (Table 2)^56^.

**Table 2:** Summary of data records.

Dataset	Data format	Deposited DB	Accession number
Illumina_Genome	fastq	NCBI SRA	PRJNA973122
Nanopore_Genome	fastq	NCBI SRA	PRJNA973122
Illumina_HiC	fastq	NCBI SRA	PRJNA973122
Illumina_Transcriptome	fastq	NCBI SRA	PRJNA973122
Genome_assembly	fasta	NCBI Genome	JAUPTC000000000
Gene_annotation	GFF3, fasta	Zenodo	7750935
TE_annotation	GFF3, fasta	Zenodo	7750935
Gene_expression_matrix	text	Zenodo	7750935

The genome assembly of *S. flavescens* has been deposited into NCBI Datasets Genome, and can be accessed according to accession number: JAUPTC000000000^57^.

In addition, gene and TE annotations, as well as gene expression matrix in three tissues (leaves, stems and roots), have been deposited into Zenodo and can be accessed according to 10.5281/zenodo.7750935 (Table 2)^58^.

The characterised transcripts/proteins involved in the biosynthesis of alkaloids or flavonoids/isoflavonoids in *S. flavescens* can be found in the gene annotation file deposited in Zenodo according to their transcript IDs shown in Table 3^58^.

**Table 3:** Transcripts involved in the biosynthesis of alkaloids or flavonoids/isoflavonoids in *S. flavescens* genome.

Gene symbol	Protein	Transcript_ID	Biosynthesis
LDC	lysine/ornithine decarboxylase	Sfla_6G0380600	alkaloids
CuAO	copper amine oxidase	Sfla_3G0368700	alkaloids
CuAO	copper amine oxidase	Sfla_3G0112200	alkaloids
CuAO	copper amine oxidase	Sfla_3G0112300	alkaloids
CuAO	copper amine oxidase	Sfla_3G0494100	alkaloids
CuAO	copper amine oxidase	Sfla_6G0426700	alkaloids
CuAO	copper amine oxidase	Sfla_7G0378200	alkaloids
CuAO	copper amine oxidase	Sfla_7G0396900	alkaloids
CuAO	copper amine oxidase	Sfla_8G0332900	alkaloids
CuAO	copper amine oxidase	Sfla_8G0131000	alkaloids
CuAO	copper amine oxidase	Sfla_8G0332200	alkaloids
CuAO	copper amine oxidase	Sfla_8G0332300	alkaloids
PAL	phenylalanine ammonia-lyase	Sfla_2G0073100	flavonoids/isoflavonoids
C4H	trans-cinnamate 4-monooxygenase	Sfla_4G0600200	flavonoids/isoflavonoids
CHS	chalcone synthase	Sfla_3G0018600	flavonoids/isoflavonoids
CHI	chalcone isomerase	Sfla_9G0118900	flavonoids/isoflavonoids
IFS	2-hydroxyisoflavanone synthase	Sfla_3G0554200	flavonoids/isoflavonoids
HIDH	2-hydroxyisoflavanone dehydratase	Sfla_3G0382900	flavonoids/isoflavonoids
HI4′OMT	isoflavone 4′-O-methyltransferase	Sfla_3G0554300	flavonoids/isoflavonoids
I3′H	isoflavone 3′-hydroxylase	Sfla_2G0433300	flavonoids/isoflavonoids
I2′H	isoflavone 2′-hydroxylase	Sfla_1G0322400	flavonoids/isoflavonoids
IFR	2′-hydroxyisoflavone reductase	Sfla_3G0069300	flavonoids/isoflavonoids
SOR	sophorol reductase /	Sfla_2G0308000	flavonoids/isoflavonoids
IF7GT	isoflavone 7-O-glucosyltransferase	Sfla_2G0636100	flavonoids/isoflavonoids
F3H	flavanone 3-hydroxylase	Sfla_3G0619300	flavonoids/isoflavonoids
FLS	flavonol synthase	Sfla_2G0632200	flavonoids/isoflavonoids
F3′5′H	flavonoid 3′, 5′-hydroxylase	Sfla_7G0366300	flavonoids/isoflavonoids
UF3GT	flavonol 3-O-glucosyltransferase	Sfla_4G0492100	flavonoids/isoflavonoids
FG2	flavonol-3-O-glucoside L-rhamnosyltransferase	Sfla_6G0522800	flavonoids/isoflavonoids
DFR	dihydroflavonol 4-reductase	Sfla_5G0056100	flavonoids/isoflavonoids
ANS	anthocyanidin synthase	Sfla_3G0033300	flavonoids/isoflavonoids
ANR	anthocyanidin reductase	Sfla_1G0406900	flavonoids/isoflavonoids
LAR	leucoanthocyanidin reductase	Sfla_4G0142000	flavonoids/isoflavonoids

## Technical Validation

### Quality control for sequencing data

For Illumina DNA sequencing data, after we removed adaptors and low quality sequences (quality score < 20), we were still able to get 99.46% of the raw reads as high quality reads, representing a depth of ~107.47 times of the genome coverage (Supplementary Table 7). For Illumina HiC DNA sequencing data, after we removed adaptors and low quality sequences (quality score < 20), we had 95.36% of the raw sequencing reads left as high quality reads, which was ~110.18 times of the genome coverage (Supplementary Table 7). For the Nanopore DNA sequencing data, the N50 of the reads was more than 25 Kb, and the longest read is more than 219 Kb (Supplementary Table 7). For the transcriptome data, after removal of adaptor and low quality sequences (quality score < 20), we had 97.00%, 96.89%, 96.19% of raw reads left as high quality reads for leaf, root and stem tissues respectively. All of these statistics indicated that these sequencing datasets are of high quality and reliability for the genome assembly study.

### Genome assembly quality assessment

The quality of our *S. flavescens* genome assembly was assessed according to the three Cs criterion: Contiguity, Completeness and Correctness. The N50 of the genome assembly is larger than 233 Mb. The contact map of Hi-C interaction for our *S. flavescens* genome assembly revealed nine chromosomal level scaffolds, which is consistent with the reported *S. flavescens* karyotype (Fig. 1d)^30^, indicating the high contiguity of the genome assembly. With respect to the completeness of the genome assembly, the BUSCO analysis with lineage “fabales_odb10” showed that 93.3% of Fabales gene orthologs could be identified in this *S. flavescens* genome assembly, including complete and fragment percentages of 91.3% and 2.0%, respectively (Table 1). For the assessment of the correctness of the genome assembly, we re-aligned clean Illumina DNA sequencing data (with adaptors and low quality sequences filtered) against the assembly using BWA (v0.7.17-r1188)^59^, and 99.72% reads could be successfully mapped, including 98.39% unique mapping and 1.33% multiple mapping respectively. The alignment of RNA-Seqs against the genome assembly showed that 97.13%, 93.93% and 96.99% reads from leaf, root and stem tissues could be successfully mapped to the genome assembly respectively (Supplementary Table 7). All these statistics and above-mentioned phylogenomics analysis as well as successful characterisation of biosynthesis genes indicated that this *S. flavescens* genome is of high quality .

**Table 4:** List of used tools/softwares.

Name	Version	Analyses	Link
Trimmomatic	v0.39	Quality control	http://www.usadellab.org/cms/?page=trimmomatic
jellyfish	v2.3.0	Genome survey	https://github.com/gmarcais/Jellyfish
GenomeScope	v1.0	Genome survey	http://qb.cshl.edu/genomescope/
FMLRC	v1.0.0	Error correction	https://github.com/holtjma/fmlrc
CANU	v2.0	Error correction, Assembly	https://github.com/marbl/canu
Raven	v1.1.10	Assembly	https://github.com/lbcb-sci/raven
SMARTdenovo	v1.0	Assembly	https://github.com/ruanjue/smartdenovo
wtdbg2	v1.1	Assembly	https://github.com/ruanjue/wtdbg2
Flye	v2.7.1	Assembly	https://github.com/fenderglass/Flye
purge_haplotigs	v1.1.1	Assembly	https://bitbucket.org/mroachawri/purge_haplotigs
minimap2	v2.17	Polishing	https://github.com/lh3/minimap2
Racon	v1.4.16	Polishing	https://github.com/isovic/racon
medaka	v1.0.3	Polishing	https://github.com/nanoporetech/medaka
nextpolish	v1.2.4	Polishing	https://github.com/Nextomics/NextPolish
3D-DNA	v180922	Scaffolding	https://github.com/aidenlab/3d-dna
Juicebox	v1.13.01	Scaffolding	https://github.com/aidenlab/Juicebox
BWA	v0.7.17-r1188	Assessment	https://github.com/lh3/bwa
BUSCO	v4.1.4	Assessment	https://busco.ezlab.org/
STAR	v2.7.8a	Assessment	https://github.com/alexdobin/STAR
Maker	v3.01.03	Annotation	https://www.yandell-lab.org/software/maker.html
StringTie	V2.1.4	Annotation	https://ccb.jhu.edu/software/stringtie/
Augustus	v3.2.3	Annotation	https://bioinf.uni-greifswald.de/augustus/
EDTA	v1.9.7	Annotation	https://github.com/oushujun/EDTA
OrthoFinder	v2.5.2	Phylogenomics	https://github.com/davidemms/OrthoFinder
CAFE	v4.2.1	Phylogenomics	https://github.com/hahnlab/CAFE
DupGen_finder	NA	Phylogenomics	https://github.com/qiao-xin/DupGen_finder
MCScanX	NA	Phylogenomics	https://github.com/wyp1125/MCScanX
SynVisio	online	Phylogenomics	https://github.com/kiranbandi/synvisio

### Supplementary information

Genomic statistics of draft assemblies from 17 different strategies.

Summary of TE annotation.

Genome information of 25 plant species used in phylogenomics analysis.

Summary of gene expansion/contraction analysis.

Duplicated gene pairs.

Genes/proteins involved in the biosynthesis of flavonoids/isoflavonoids.

Summary of sequencing data in this project.

## Data Availability

The information for all bioinformatics tools used in this study is listed in Table 4. All code/scripts used for the genome assembly and analyses can be accessed on GitHub (https://github.com/zpqu/KS_WGS_scripts).
